# Pediatric-Onset Multiple Sclerosis at Age 10 Following Nephrotic Syndrome: Early Recognition and Successful Treatment With Fingolimod

**DOI:** 10.7759/cureus.103071

**Published:** 2026-02-05

**Authors:** Imane Mezdaoui, Khadija Mouaddine, Chaimae Nahi, Bouchra Chkirate

**Affiliations:** 1 Pediatrics, Rabat Children Hospital, Rabat, MAR; 2 Pediatric Rheumatology, Rabat Children Hospital, Rabat, MAR

**Keywords:** early-onset multiple sclerosis, fingolimod, neurology, ophthalmology, pediatric multiple sclerosis

## Abstract

Pediatric-onset multiple sclerosis before the age of 10 is rare and poses significant diagnostic challenges. We report a 10-year-old boy who developed multiple sclerosis five years after remission of nephrotic syndrome. He presented with progressive left eye visual loss and vertigo. Advanced magnetic resonance imaging (MRI) revealed demyelinating lesions with a central vein sign and paramagnetic rim, emerging biomarkers that support the diagnosis of pediatric multiple sclerosis. Cerebrospinal fluid analysis demonstrated type 2 oligoclonal bands, while anti-aquaporin-4 and anti-myelin oligodendrocyte glycoprotein antibodies were negative. The diagnosis of multiple sclerosis was established according to the 2017 McDonald criteria. Early treatment with fingolimod (0.5 mg daily) resulted in complete clinical and radiological disease suppression over an 18-month follow-up period. This case highlights the value of advanced MRI biomarkers in very early-onset multiple sclerosis, the importance of systematically excluding disease mimics in prepubertal children, and the effectiveness of early initiation of high-efficacy disease-modifying therapy. Early recognition and prompt treatment are essential to optimize outcomes in pediatric multiple sclerosis.

## Introduction

Pediatric-onset multiple sclerosis (POMS) accounts for 3%-10% of multiple sclerosis (MS) cases, with onset before age 10 occurring in only 0.2%-0.7% [1-3]. This rarity creates diagnostic uncertainty, as acute disseminated encephalomyelitis (ADEM) is far more common in prepubertal children [4]. Other important mimics in this age group include neuromyelitis optica spectrum disorder (NMOSD), myelin oligodendrocyte glycoprotein (MOG) antibody-associated disease, infectious or post-infectious demyelination, and metabolic or genetic leukodystrophies. Recent advances in magnetic resonance imaging (MRI) biomarkers, including the central vein sign and paramagnetic rim lesions, show promise in distinguishing MS from mimics, though pediatric data remain limited [5,6]. Evidence increasingly supports early high-efficacy disease-modifying therapy (DMT) in POMS [7-10]. We report successful early diagnosis and treatment of MS in a 10-year-old boy, demonstrating practical application of advanced diagnostic techniques and contemporary treatment paradigms.

## Case presentation

A 10-year-old boy presented with a six-month history of progressive left eye vision loss culminating in light perception only, accompanied by recurrent vertigo. Medical history included nephrotic syndrome at age 4, successfully treated with corticosteroids, achieving remission by age 5. No family history of MS or autoimmune diseases existed. Birth history and developmental milestones were normal.

Neurological examination revealed an alert mental status with age-appropriate cognition. Cranial nerve assessment showed left optic nerve dysfunction with divergent strabismus. Motor examination demonstrated normal tone and 5/5 strength bilaterally. Sensory examination, reflexes, coordination, and gait were normal.

Ophthalmological evaluation revealed right eye visual acuity of 10/10 and left eye light perception only. Left relative afferent pupillary defect was present. Fundoscopy demonstrated right papillary hyperemia and complete left optic atrophy.

**Figure 1:**
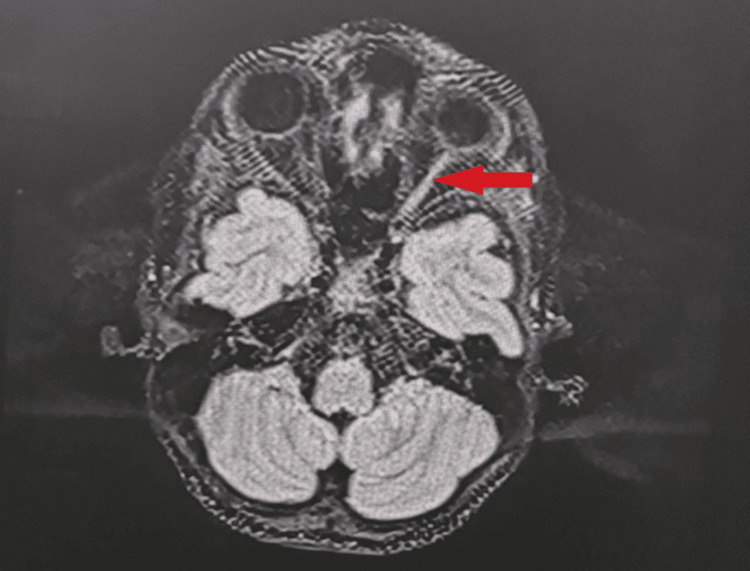
Axial T2-weighted brain MRI demonstrating left optic nerve involvement. The left optic nerve shows a hyperintense signal (red arrow) consistent with optic neuritis. The asymmetric optic nerve hyperintensity corresponds to the patient’s complete left eye visual loss and subsequent optic atrophy. MRI: magnetic resonance imaging

**Figure 2:**
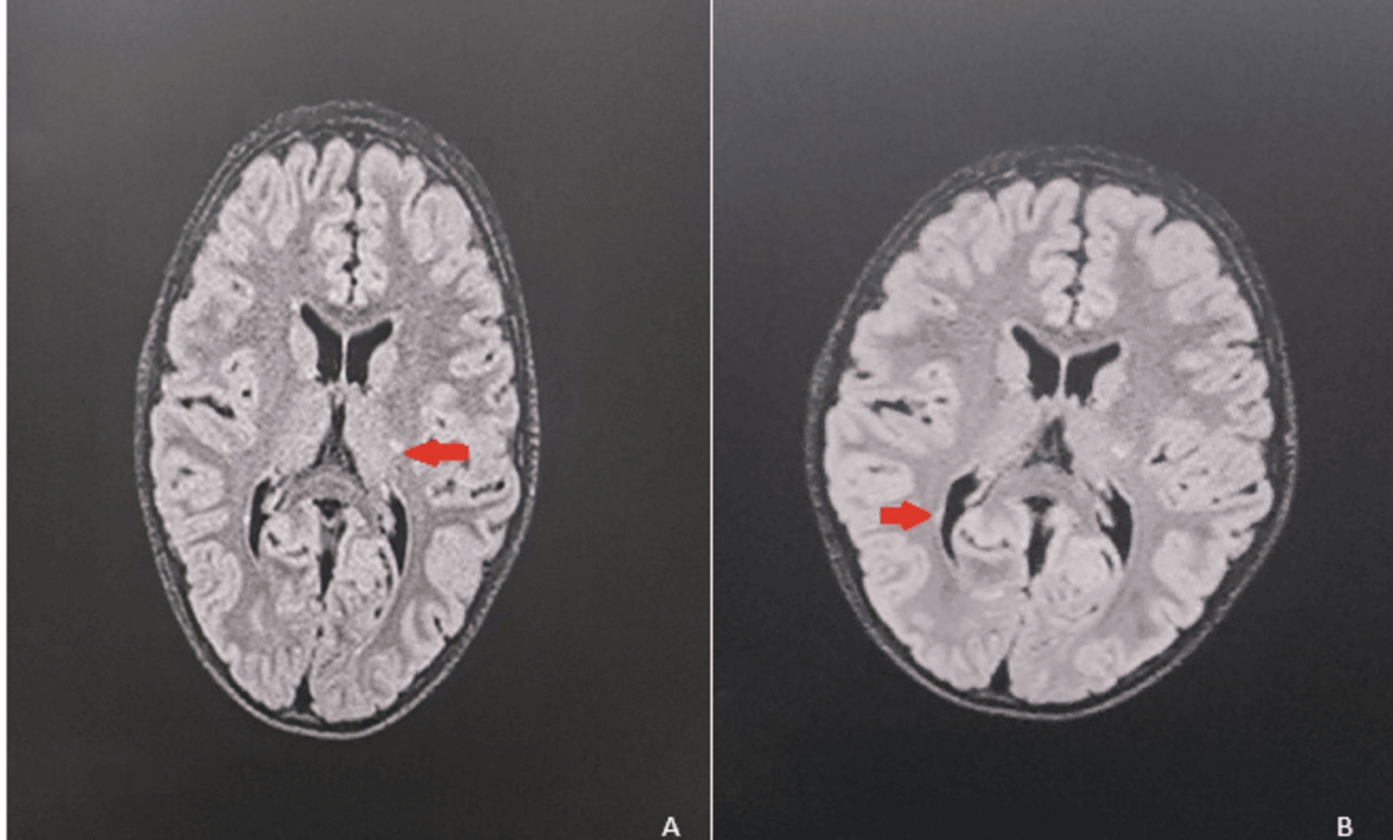
Axial T2/FLAIR brain MRI showing periventricular demyelinating lesions. (A) Left periventricular white matter lesion in the region of the posterior internal capsule adjacent to the left lateral ventricle (red arrow). (B) Additional periventricular demyelinating lesion (red arrow) with characteristic features of pediatric multiple sclerosis, including periventricular distribution and T2 hyperintensity. MRI: magnetic resonance imaging; FLAIR: fluid-attenuated inversion recovery

Brain and cervical spine MRI revealed extensive T2/fluid-attenuated inversion recovery (FLAIR) hyperintensity throughout the left retrobulbar optic nerve extending to the chiasm without gadolinium enhancement (Figure 1). Periventricular lesions included a lesion at the left posterior internal capsule demonstrating the central vein sign on susceptibility-weighted imaging (SWAN sequence) and paramagnetic rim on phase sequence, indicating chronic active inflammation. Additional demyelinating lesions were identified in the left thalamus and left pons (Figure 2).

Cerebrospinal fluid (CSF) analysis showed type 2 oligoclonal bands (CSF-positive, serum-negative), IgG index 0.577, mild lymphocytic pleocytosis, and normal protein. Anti-aquaporin-4 and anti-MOG antibodies were negative. Complete blood count (CBC), metabolic panel, and rheumatologic studies were normal.

ADEM was excluded due to the absence of encephalopathy and progressive course. NMOSD was excluded by negative aquaporin-4 antibodies. MOG antibody-associated disease (MOGAD) was unlikely given negative MOG antibodies and oligoclonal bands (present in only ~10% of MOGAD) [2,4]. MS was supported by the McDonald 2017 criteria: dissemination in space (optic nerve, periventricular, thalamus, and brainstem) and time (CSF oligoclonal bands, progressive course).

Advanced MRI biomarkers strengthened diagnosis. The central vein sign demonstrates 80%-95% sensitivity for MS versus 0%-15% in mimics [2,3]. Paramagnetic rim indicates chronic active inflammation and predicts aggressive disease [2,3].

High-risk features (age 10, permanent deficit, and paramagnetic rim) warranted early high-efficacy therapy. Fingolimod 0.5 mg daily was initiated with first-dose cardiac monitoring showing no adverse events. Monitoring protocol included monthly CBC initially, quarterly liver function tests (LFTs), biannual ophthalmology exams, and 6-12 monthly MRIs.

Over the 18-month follow-up, there were zero relapses, no new MRI lesions, expected mild lymphopenia, normal LFTs, and no macular edema. Visual acuity remained stable. The Expanded Disability Status Scale (EDSS) remained 0 (excluding visual deficit). Excellent adherence was maintained.

## Discussion

Advanced MRI biomarkers proved valuable. The central vein sign helped distinguish MS from ADEM [5,6]. Paramagnetic rim indicated chronic active inflammation, justifying high-efficacy therapy [6,7]. These biomarkers remain underutilized in pediatric populations.

Prepubertal MS shows unique CSF characteristics with oligoclonal bands sometimes initially absent [1,11]. Our patient's borderline IgG index but positive oligoclonal bands emphasizes isoelectric focusing superiority over index calculation in young children.

Systematic exclusion of mimics is critical. ADEM, 10-fold more common in children < 10 years [4,12], was excluded by the absence of encephalopathy [1,5]. MOGAD was unlikely given negative antibodies and oligoclonal bands [4,5,12]. NMOSD was excluded by negative aquaporin-4 antibodies [1,5]. Recent studies show declining POMS diagnoses in children < 12 years, likely due to MOGAD recognition [12], underscoring antibody testing importance.

The incidental syringomyelic cavity at C4-C5 remained stable, confirming it was unrelated to MS. This emphasizes distinguishing MS pathology from incidental findings.

Our approach reflects evidence favoring early high-efficacy therapy. The PARADIGMS trial demonstrated fingolimod's superiority over interferon beta-1a (82% relapse reduction) [7,8]. Real-world data show 86% of children on moderate-efficacy therapy discontinue by five years versus 51% on high-efficacy therapy [9,10]. Our patient's response-zero relapses and no MRI activity over 18 months-corroborates fingolimod's effectiveness. Paramagnetic rim presence justified early aggressive treatment.

The relationship between nephrotic syndrome and MS remains unclear. Both involve immune dysregulation with shared HLA associations [1,13-16]. However, the five-year interval suggests coincidental phenomena. Prospective studies examining autoimmune clustering are needed.

This case provides actionable insights: maintain diagnostic suspicion for MS in prepubertal children with nonencephalopathic demyelinating events, utilize advanced MRI biomarkers when available, systematically exclude mimics through antibody testing, consider early high-efficacy DMT with high-risk features, and implement comprehensive monitoring.

A longer follow-up is needed for treatment durability and long-term safety assessment. Fingolimod's effects on growth, development, and infection risk require surveillance. Cognitive outcomes, affected in 30%-50% of pediatric MS patients [1,13-16], necessitate serial neuropsychological testing.

## Conclusions

This case demonstrates the successful application of contemporary approaches in a 10-year-old boy with MS. Advanced MRI biomarkers facilitated diagnosis, systematic testing excluded mimics, and early fingolimod achieved excellent control. These findings support early high-efficacy treatment in pediatric MS, particularly with aggressive features including young age, permanent deficits, and paramagnetic rim lesions. Clinicians can apply these strategies to optimize outcomes in prepubertal MS.
